# Arrhythmogenic Effects of Genetic Mutations Affecting Potassium Channels in Human Atrial Fibrillation: A Simulation Study

**DOI:** 10.3389/fphys.2021.681943

**Published:** 2021-05-31

**Authors:** Rebecca Belletti, Lucia Romero, Laura Martinez-Mateu, Elizabeth M. Cherry, Flavio H. Fenton, Javier Saiz

**Affiliations:** ^1^Centro de Investigación e Innovación en Bioingeniería, Universitat Politècnica de València, Valencia, Spain; ^2^Departamento de Teoría de la Señal y Comunicaciones y Sistemas Telemáticos y Computación, Universidad Rey Juan Carlos, Madrid, Spain; ^3^School of Computational Science and Engineering, Georgia Institute of Technology, Atlanta, GA, United States; ^4^School of Physics, Georgia Institute of Technology, Atlanta, GA, United States

**Keywords:** genetic mutations, *in silico* modeling, atrial fibrillation, potassium channels, channelopathy

## Abstract

Genetic mutations in genes encoding for potassium channel protein structures have been recently associated with episodes of atrial fibrillation in asymptomatic patients. The aim of this study is to investigate the potential arrhythmogenicity of three gain-of-function mutations related to atrial fibrillation—namely, KCNH2 T895M, KCNH2 T436M, and KCNE3-V17M—using modeling and simulation of the electrophysiological activity of the heart. A genetic algorithm was used to tune the parameters’ value of the original ionic currents to reproduce the alterations experimentally observed caused by the mutations. The effects on action potentials, ionic currents, and restitution properties were analyzed using versions of the Courtemanche human atrial myocyte model in different tissues: pulmonary vein, right, and left atrium. Atrial susceptibility of the tissues to spiral wave generation was also investigated studying the temporal vulnerability. The presence of the three mutations resulted in an overall more arrhythmogenic substrate. Higher current density, action potential duration shortening, and flattening of the restitution curves were the major effects of the three mutations at the single-cell level. The genetic mutations at the tissue level induced a higher temporal vulnerability to the rotor’s initiation and progression, by sustaining spiral waves that perpetuate until the end of the simulation. The mutation with the highest pro-arrhythmic effects, exhibiting the widest sustained VW and the smallest meandering rotor’s tip areas, was KCNE3-V17M. Moreover, the increased susceptibility to arrhythmias and rotor’s stability was tissue-dependent. Pulmonary vein tissues were more prone to rotor’s initiation, while in left atrium tissues rotors were more easily sustained. Re-entries were also progressively more stable in pulmonary vein tissue, followed by the left atrium, and finally the right atrium. The presence of the genetic mutations increased the susceptibility to arrhythmias by promoting the rotor’s initiation and maintenance. The study provides useful insights into the mechanisms underlying fibrillatory events caused by KCNH2 T895M, KCNH2 T436M, and KCNE3-V17M and might aid the planning of patient-specific targeted therapies.

## Introduction

Atrial fibrillation (AF) is the most common sustained cardiac arrhythmia and it is characterized by disorganized rapid electrical activations of the upper chambers of the heart. It affects almost 2% of the general population and its incidence increases with age. Even if the arrhythmia itself does not lead to sudden death, it is associated with increased mortality and increased risk (from three to fivefold) of thromboembolic stroke and heart failure. Associated co-morbidities and risk factors are cardiovascular diseases, endocrine, and metabolic disorders, as well as respiratory conditions and lifestyle factors (obesity, smoking, and alcoholism…) (Kornej et al., 2020). In 30% of cases, however, the arrhythmia manifests in asymptomatic subjects, not affected by any of the previous pathologies. This form of AF is known as “Lone AF” and it has been related to genome alterations that provoke the outbreak of arrhythmic episodes (Chugh et al., 2014). Extensive research has investigated the association of fibrillatory events with a genetic predisposition, holding inheritance responsible for the onset of such a pathology (Fatkin et al., 2007; Fox et al., 2004; Andreasen et al., 2015; Feghaly et al., 2018; Ragab et al., 2020). The role of genetic mutations affecting genes that encode ion channel protein structures on the genesis of arrhythmias has become a matter of intense study in the past few decades.

In particular, many studies have focused on the effects of genetic mutations that affect genes encoding potassium channels and causing serious forms of arrhythmia. In these works, some of the mutations shared different phenotypes such as long- and short-QT syndromes, sudden death, Brugada syndrome, bradycardia, and also familial AF (Chen et al., 2003; Hong et al., 2005b; Lundby et al., 2007; Das et al., 2009; Bartos et al., 2011, 2013; Hattori et al., 2012; Henrion et al., 2012; Christophersen et al., 2013; Ki et al., 2014; Wang et al., 2014; Steffensen et al., 2015). In fact, rapid and slow rectifier potassium currents are the main components of the repolarization phase in cardiac myocyte’s electrophysiology (Rudy, 2009). Therefore, genetic defects impairing the normal function of these channels could lead to abnormal conduction of currents which, in turn, could reflect in shortening or lengthening of the action potential duration at 90% of repolarization (APD_90_), known to be pro-arrhythmic (Chen et al., 2003; Hong et al., 2005a; Christophersen et al., 2013). In this framework, computational modeling was demonstrated to be a valuable tool in characterizing AF pathophysiology and in reproducing the dynamics behind AF outbreaks (Dössel et al., 2012). Some of the latest rare variants reported in the literature and affecting the rapid component of the potassium channels are T895M, T436M, and KCNE3-V17M. The first two mutations were found and characterized by Hayashi et al. (2015), and affected the KCNH2 (hERG) gene, which encodes the α-subunit of the channel complex Kv11.1, underlying the I_*Kr*_ current. The third mutation was experimentally investigated by Lundby et al. (2008) and impaired the KCNE3 gene, which is responsible for the coding of accessory subunits of the Kv channels, underlying I_*Kr*_ and I_*to*_ currents.

In this work, we investigate the effects of the three above-mentioned genetic gain-of-function missense mutations using human atrial mathematical models to reproduce healthy and pathological conditions in three different heart regions: right atrium (RA), left atrium (LA), and pulmonary vein (PV). The computational approach we use aims at reproducing and characterizing the dynamics induced by the mutations at the cellular and tissue scales. Therefore, AF-related parameters were extracted and analyzed from single-cell and tissue simulations to evaluate the mutations’ potential to generate a substrate prone to arrhythmias.

## Materials and Methods

### Experimental Data

Mutant experimental data of the three selected mutations appeared in two previous studies found in the literature (Lundby et al., 2008; Hayashi et al., 2015), where lone AF patients with a family history were investigated through genome screening to find rare variants in genes associated to AF. In the first study (Hayashi et al., 2015), two genetic mutations, T895M and T436M, were reported. They affected the gene KCNH2, which encodes the human heter-a-go-go protein forming the α-subunit of the rapid potassium channel. In the case of the KCNH2 T895M mutation, the proband was an adult male patient with persistent AF who underwent a cardiac ablation procedure. Experimental data showed significantly higher peak and tail current densities and an increase in the values of the time constant at −40 and −30 mV. The patient found with the KCNH2 T436M mutation was instead suffering from persistent AF and presented higher values for peak current density and time constants. In the second study (Lundby et al., 2008), the mutation V17M was found in an adult male with lone AF and it affected the gene KCNE3. This gene is responsible for encoding an accessory subunit of Kv channels. The mutation, in fact, provoked the impairment not only of Kv11.1, but also of Kv4.3 channel complexes, so basically affecting the current flowing in the I_*Kr*_ and I_*to*_ channels. Their respective electrophysiological studies showed that Kv11.1/KCNE3-V17M double the time constant at −120 mV with respect to Kv11.1/KCNE3, and that the Kv4.3/KCNE3-V17M steady-state inactivation curves shifted of 14 mV toward more positive potentials with respect to Kv4.3/KCNE3. Peak currents for both Kv11.1 and Kv4.3 were highly enhanced by the mutation.

### Parameterization of Potassium Currents

The Hodgkin-Huxley formulations of the WT rapid delayed rectifier potassium current I_*Kr*_ and of the transient outward potassium current I_*to*_ were adopted from the Courtemanche et al. (1998) model. These equations were modified by adding additional parameters in order to reproduce the specific changes in the channels’ dynamics provoked by the effect of the genetic mutations under study. In particular, the behavior of the gates was modified by including parameters in the forward (α) and backward (β) rate constants. The magnitude of the rates was altered by the parameters p_0r_, p_0t_, p_3r_, and p_3t_, which are scaling factors. The voltage dependence of the rates was modified by the parameters p_1r_, p_1t_, p_4r_, and p_4t_, which are voltage shifts, and by the parameters p_2r_, p_2t_, p_5r_, and p_5t_, which change the slope of the sigmoid curves. Parameters p_6r_ and p_6t_ are scaling factors applied to the I_*Kr*_ and I_*to*_ channel conductance and, finally, parameters p_7r_ and p_8r_ affect the instantaneous inactivation formulation of I_*Kr*_ (voltage shift and change in the slope, respectively). As shown in equations (1) to (6), nine parameters (p_0r_ to p_8r_) were included in the I_*Kr*_ formulation, and seven parameters (p_0t_ to p_6t_) were included in the I_*to*_ inactivation gates and current formulation.

(1) $$\alpha_{x\,r}=p_{0\,r}\cdot 0.0003\,\frac{V+14.1+p_{1\,r}}{1-{\text{e}}^{\frac{V+14.1+p_{1\,r}}{-5\cdot p_{2\,r}}}}$$

(2) $$\beta_{x\,r}=p_{3\,r}\cdot 7.3898\cdot 10^{-5}\,\frac{V-3.3328+p_{4\,r}}{e^{\frac{V-3.3328+p_{4\,r}}{5.1237\cdot p_{5\,r}}}-1}$$

(3) $$I_{K\,r}=C_{m}\cdot p_{6\,r}\cdot g_{K\,r}\cdot x\,r\,\frac{V-E\,k}{1+{\text{e}}^{\frac{V+15+p_{7\,r}}{22.4\cdot p_{8\,r}}}}$$

(4) $$\alpha_{o\,i}=\frac{p_{0\,t}}{18.53\,{\text{e}}^{\frac{V+113.7+p_{1\,t}}{10.95\cdot p_{2\,t}}}}$$

(5) $$\beta_{o\,i}=\frac{p_{3\,t}}{35.56\,{\text{e}}^{\frac{V+1.26+p_{4\,t}}{-7.44\cdot p_{5\,t}}}}$$

(6) $$I_{t\,o}=C_{m}\cdot p_{6\,t}\cdot g_{t\,o}\cdot o\,i\cdot o\,a^{3}\,\left(V-E\,k\right)$$

### Sensitivity Analysis

The two sets of proposed parameters underwent a one-at-a-time (OAT) sensitivity analysis to evaluate their influence on the outcomes of interest. Therefore, activating and tail currents, time constants and inactivation curves were computed for each mutation varying one parameter at a time and varying its value from 0.1 to 5, in steps of 0.1. This range of values was only selected to detect those with the highest influence on the electrophysiological characteristics of the currents. This analysis is relevant to identify the parameters to be fitted to better reproduce the alterations that the mutations produce on the dynamics of the channels, whose values were not constrained to the abovementioned range. Results of sensitivity analysis are shown in Supplementary Figures 1–3.

### Model Parameters Estimation

The estimation of model parameter values was performed using a genetic algorithm (Cairns et al., 2017), implemented in Python, with an initial population size of 1,000 individuals and 20 generations. The values of the parameters were left free to vary without constraining them into the range used for the sensitivity analysis. Parallelization was implemented to improve computational performances. Genetic algorithms are robust global search heuristic algorithms and have a demonstrated efficiency in finding good combinations of parameters in some previous cardiac reparameterization problems (Syed et al., 2005; Bot et al., 2012; Groenendaal et al., 2015; Cairns et al., 2017; Smirnov et al., 2020). The main advantages in using such an algorithm are high independence from the initial guess and a low chance to be stuck at a local minimum, thus converging prematurely to a non-optimal solution. As the algorithm was launched, an initial population of 1,000 individuals was randomly generated from a uniform distribution of samples, creating the first generation. Each individual was evaluated using a specifically designed fitness function, and a score value was calculated. Then, individuals were sorted depending on their score value and “Selection,” “Crossover,” and “Mutation” procedures took place. Different configurations of methods were evaluated before running the final simulations in order to find the best combination of techniques. For the Selection procedure, the “Simple Select” method was applied: parents for the next generation were randomly selected from the best fit individuals. The self-adaptive Simulated Binary Crossover (SBX) procedure was implemented since it proved to have better performance at the mating stage (Deb and Kumar, 1995; Deb et al., 2007): the parameter η_*c*_, in fact, guarantees diversity in the offspring solution and adapts based on the parents’ fitness scores. It is contracted or expanded if children have better or worse score values, respectively. While crossovers have control in exploiting an area of the parameter space to find the best solution, the mutation is important for the exploration of new areas. In this study, a gaussian mutation operator was chosen with a mutation rate set at 0.9. Finally, since crossover and mutation guarantee a good exploration and exploitation of search space, elitism seemed unnecessary to ensure the algorithm did not “spoil” a good solution. Thus, the elitism percentage was set to 0. The reduction of dimensionality, and therefore complexity, of the search space through previous sensitivity analysis played an important role in terms of reduction of computational resources and complexity of the genetic algorithm. Finally, the fitness function employed to evaluate individuals was defined as the weighted sum of root-mean-square error between simulated and experimental data.

### Atrial Action Potential Model

Action potential simulations were carried out using versions of the human atrial myocyte model proposed by Courtemanche et al. (1998), which was previously modified to also include the formulation of the acetylcholine-activated K^+^ current (I_*KACh*_) proposed by Grandi et al. (2011) the acetylcholine concentration was set to 0.005 μM, which is within the physiological range. Such a current allowed the integration of the autonomic nervous system influence, in particular its parasympathetic stimulations, on atria electrophysiology. In fact, it plays an important role in the repolarization phase of the action potential by reducing its duration, both in physiological and pathological conditions (Bosch et al., 1999; Dobrev et al., 2002). The presence of the mutations was simulated by including the I_*Kr*_ and I_*to*_ alterations optimized at the ionic channel level. Furthermore, atrial heterogeneities were taken into account by modifying the channel’s conductances using the scale factors reported in Table 1, similarly to Martinez-Mateu et al. (2018). This allowed the simulation of three different atrial regions: right atrium (RA), left atrium (LA), and pulmonary vein (PV). The combination of wild-type and the three mutations with the three atrial regions resulted in 12 atrial models, namely, WT, KCNH2 T895M, KCNH2 T436M, and KCNE3-V17M in the three tissues RA, LA, and PV.

**TABLE 1:** Scaling factors of the channels’ conductances to reproduce atrial heterogeneities.

	**RA**	**LA**	**PV**
I_*CaL*_	1	0.9	0.8
I_*Kr*_	1	2	2.5
I_*KACh*_	1	1	1
I_*to*_	1	1	0.9
I_*K1*_	1	1	0.9
I_*Ks*_	1	1	1.9

### Single Cell Simulations

Atrial action potential models were excited by a stimulus current with an amplitude of 20 pA/pF and a duration of 2 ms. The ordinary differential equations of the Courtemanche single-cell model were solved using the variable time-step solver *ode15s* in MATLAB. The virtual cells were paced for 60 s at 1 Hz for stabilization. This protocol preserved the original spike-and-dome morphology and APD_90_ value and guaranteed a stable behavior of the model in the time interval required to perform the simulations (Martinez-Mateu et al., 2018). Then, the action potentials (AP) and ionic currents elicited by the 61st pulse were analyzed. In addition, APD restitution (APDr) curves and their maximum slope values were computed in order to study the effects of the stimulation frequency in the presence of genetic mutations. For this purpose, cellular models were stimulated at basic cycle lengths (BCL) from 300 to 1,700 ms with an increment of 10 ms. APDr curves were generated by plotting the APD_90_ values of the 61st pulse as a function of the BCL.

### Tissue Simulations

The 12 versions of the Courtemanche model were incorporated into a 2-dimensional mesh, which was used to model a tissue patch of dimensions 5 cm × 5 cm with a longitudinal conductivity of 0.0022 S/cm⋅pF, an anisotropy ratio of 0.35 and a spatial resolution of 300 μm (Ferrer et al., 2015; Martinez-Mateu et al., 2018). Such dimensions were adopted for the three types of tissue to study and compare the vulnerability to re-entry generation. The monodomain formalism was solved using the Elvira software (Heidenreich et al., 2010) with a time step of 0.01 ms (Ferrer et al., 2015; Martinez-Mateu et al., 2018). Tissues were stimulated by applying a 2 ms-excitation current of amplitude 100 pA/pF.

The potential of the 12 models to initiate spiral waves was investigated by applying a cross-field protocol. Tissues were paced with 10 planar pulses (S1) for stabilization at a BCL of 1,000 ms, at the bottom edge of the tissue. Then, a rectangular S2 stimulus of dimensions 1.8 cm × 2.6 cm was applied in the left bottom corner at different S1–S2 time intervals and simulations of 5 s-duration were run. Similar protocols have been applied to study tissue vulnerability in previous studies (Kharche et al., 2008, 2012; Loewe et al., 2014; Zulfa et al., 2016). Temporal vulnerability to re-entry generation was quantified as the width of the time window during which an S2 stimulus, applied to the refractory tail of the 10th S1, initiates a re-entry completing at least two cycles around its center.

Furthermore, variations in the rotors’ lifetime were investigated. Rotors were elicited every 2 ms inside the vulnerable window (VW) and classified into three main categories based on their life span: spiral waves that were sustained for the entire simulation time (sustained VW), spiral waves that lasted between one and 5 s, and spiral waves that lasted less than 1 s.

Spatial organization and temporal information of the rotor’s dynamics were investigated by performing phase analysis on simulated AP signals, with the use of the Hilbert transform (Martinez-Mateu et al., 2018, 2019). The rotor’s tip trajectory was tracked by computing singularity points from phase maps. The stability of the re-entry was quantified in terms of life span and extension of the meandering path of the tip in the tissue. For this purpose, each rotor’s tip trajectory was surrounded by an ellipse and its area was computed.

## Results

### Mathematical Modeling of the Mutations

**FIGURE 1:**
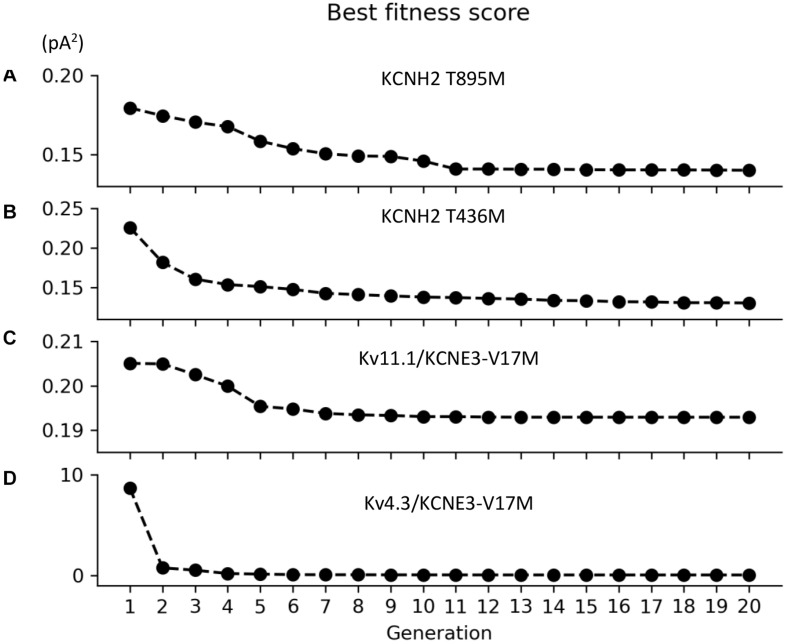
Convergence of the root-mean square value (pA^2^) of the best individuals as function of the generation for the **(A)** KCNH2 T895M, **(B)** KCNH2 T436M, **(C)** Kv11.1/KCNE3-V17M, and **(D)** Kv4.3/KCNE3-V17M mutations.

**FIGURE 2:**
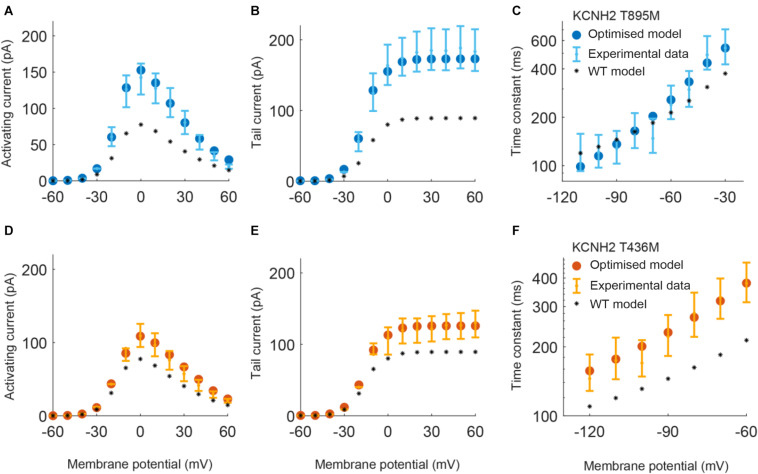
Resulting I_*Kr*_ currents and time constants obtained by using the parameters estimated with the optimization procedure of the KCNH2 T895M (blue) and KCNH2 T436M (orange) models. Error bars indicate experimental data (target) and closed circles correspond to simulation results; wild-type (WT) model results are represented by black stars. **(A,D)** Activating current. **(B,E)** Tail current. **(C,F)** Deactivation time constant.

**FIGURE 3:**
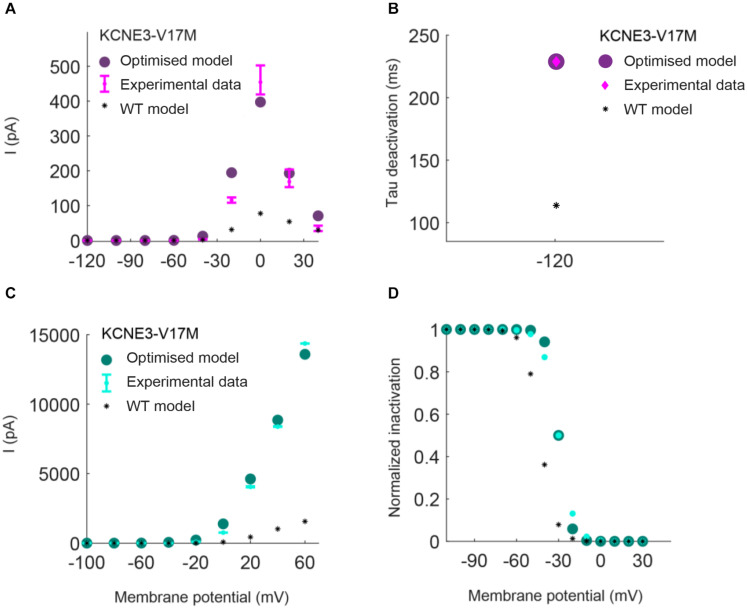
Resulting I_*Kr*_ (top) and I_*to*_ (bottom) I/V relationship and inactivation curves obtained by using the parameters estimated with the optimization procedure of the Kv11.1/KCNE3-V17M mutation (purple) and Kv4.3/KCNE3-V17M mutation (green). Error bars indicate experimental data (target) and closed circles correspond to simulation results; wild-type (WT) model results are represented by black stars. **(A,C)** I–V relationships. **(B)** Deactivation time constant at −120 mV. **(D)** Steady-state inactivation curve.

Parameters to be adjusted were selected from the initial set of proposed parameters in equations (1) to (6) based on the results of the sensitivity analysis previously performed (see Supplementary Figures 1–3). We selected the parameters p_3r_, p_4r_, p_5r_, and p_6r_ to model the alterations that the KCNH2 T895M and KCNH2 T436M mutations produce in activating and tail currents, and in the deactivation time constants, as they were the parameters influencing them the most (see Supplementary Figure 1). Despite its evident effect on deactivation time constant, the parameter p_2r_ was not taken into account because its changes would affect the activation time constant values as well and no experimental data about it was provided. The chosen parameters for the Kv11.1/KCNE3-V17M mutation were p_3r_, p_6r_, and p_8r_ as they affected the I–V curve the most and the value of the deactivation time constant at −120 mV (see Supplementary Figure 2). Finally, the selected parameters for the Kv4.3/KCNE3-V17M mutation were p_0t_, p_2t_, p_3t_, p_5t_, and p_6t_; even if some of them act similarly on the I–V curves and on the steady-state inactivation, their combined effect allowed a more precise fitting of the experimental data (see Supplementary Figure 3). Final parameters’ values are reported in Table 2. The genetic algorithm proved to be a valuable tool in tuning model parameter values. An accurate fitting of potassium channel dynamics impaired by the mutations’ effects was achieved, as demonstrated by the trend of the best fitness score over generations in Figure 1, which is monotonically decreasing. Figures 2, 3 show the optimization procedure results (closed circles) together with the target experimental data (error bars). Wild-type data are presented for comparison to the healthy case (black stars). Figures 2A–F show the activating current, the tail current, and the deactivation time constant of the KCNH2 T895M (blue) and KCNH2 T436M (orange) mutations, respectively. Figures 3A,B present the I–V relationship curve and the deactivation time constant value at −120 mV for the KCNE3-V17M mutation affecting the channel complex Kv11.1. Figures 3C,D depict the I–V relationship and the steady-state inactivation curves for Kv4.3/KCNE3-V17M.

**TABLE 2:** Parameters’ values to reproduce the alterations produced by the studied mutations.

**I_*Kr*_**				**I_*to*_**	
**Parameter**	**KCNH2 T895M**	**KCNH2 T436M**	**KCNE3-V17M**	**Parameter**	**KCNE3-V17M**
p_3r_	2.134	1.08	0.51	p_0t_	2
p_4r_	51.14	46.96	–	p_2t_	1.87
p_5r_	3.49	4.64	–	p_3t_	8.14
p_6r_	1.99	1.41	6.18	p_5t_	0.68
p_8r_	–	–	0.71	p_6t_	12.56

### Effects of the Mutations on AP Characteristics

**FIGURE 4:**
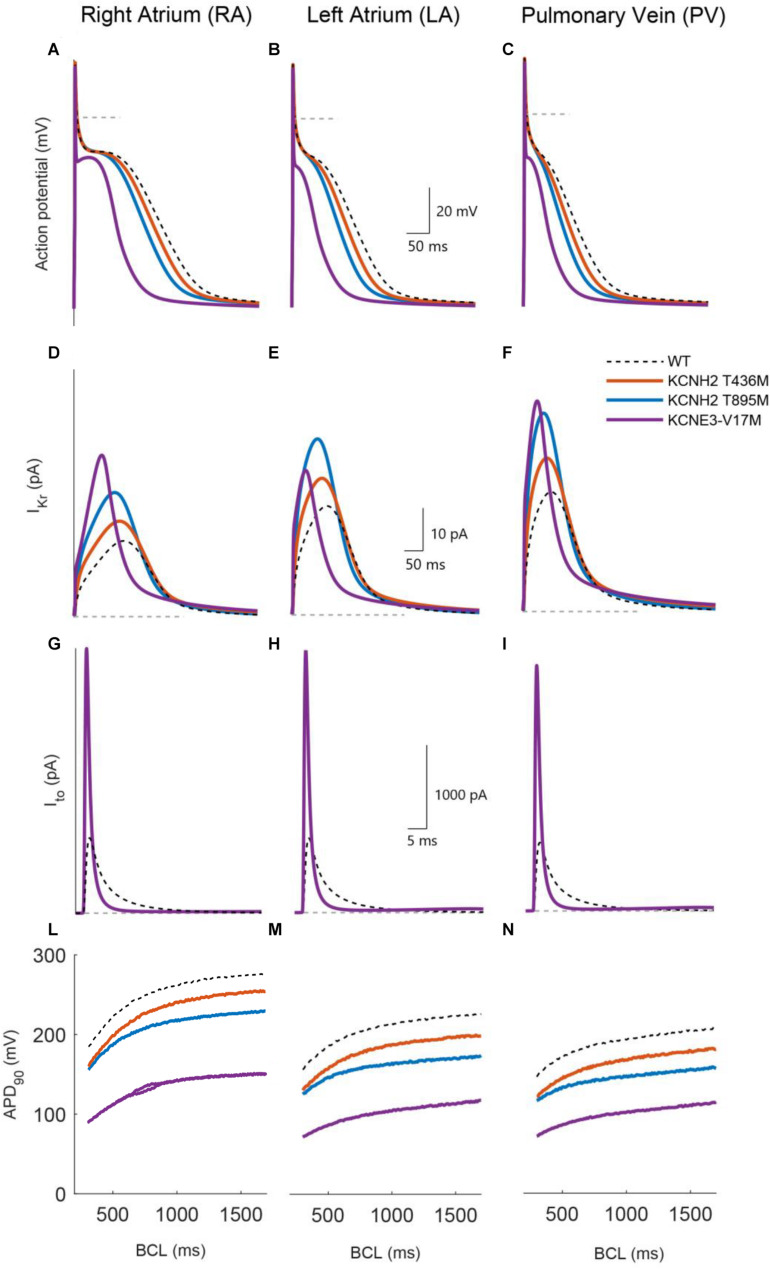
Simulated effects of the KCNH2 T895M (blue), KCNH2 T436M (orange), and KCNE3-V17M (purple) mutations in human single cell models of right atrium, left atrium, and pulmonary vein regions (from left to right). **(A–C)** Action potential. **(D–F)** Rapid K^+^ current. **(G–I)** Transient outward K^+^ current. **(L–N)** APD_90_ restitution curve. WT curves in dashed dark lines are included for comparison.

Figure 4 compares the action potentials, rapid and transient outward potassium currents, and restitution properties of the three mutations: KCNH2 T895M, KCNH2 T436M, and KCNE3-V17M (blue, orange, and purple, respectively) to the wild-type (dashed dark lines) in the three simulated tissues: RA, LA, and PV (from left to right). Figures 4A–C shows that the three mutations yielded a reduction of the APD_90_ values in all three regions under study: This is strictly related to higher I_*Kr*_ and I_*to*_ amplitudes (Figures 4D–I, respectively). Furthermore, APD restitution curves showed a flattened trend for mutations with respect to WT conditions and a shift toward smaller APD values (Figures 4L–N).

The effects of the KCNH2 T436M mutation are presented in Figure 4 by solid orange lines. Peak values of the rapid K^+^ current (Figures 4D–F) increased by 29% its WT value in RA and by 28% in the LA and PV regions. The induced APD_90_ shortened by 9% in the RA and by 13% in LA and PV (Figures 4A–C). As shown in Figures 4L–N, the shift to lower APD_90_ values is minor and restitution curve flattening was almost unaffected by the mutation; the percentage of maximum slope value decrease was only 3% in both LA and RA, while an increase of 8% was found in the PV region.

The KCNH2 T895M mutation induced an I_*Kr*_ peak increment of 63% in RA, 62% in LA, and 67% in PV region, as presented in panels D–F. The consequent APD_90_ reduction, shown in Figures 4A–C, was 18% in RA, 23% in LA, and 24% in PV. This mutation also flattened the APDr curve and produced a downward shift (Figures 4L–N). The maximum value of the restitution curve slope decreased by 22% in RA, 13% in LA, and 15% in PV.

The simulation of the KCNE3-V17M mutation increased the I_*Kr*_ current (Figures 4D–F) peak by 113% in RA, by 33% in LA, and by 76% in the PV region. The transient outward current amplitude incremented by 252% in RA, 254% in LA, and 259% in PV (Figures 4G–I). These changes in current amplitudes provoked an APD_90_ shortening of 46% in RA, 51% in LA, and 47% in PV. The APD restitution curve shifted to lower values of APD, as shown in panels L–N, and its trend presented a pronounced flattening. The maximum slope value reduced in fact by 43% in RA, by 46% in LA, and by 28% in PV, compared to the WT case. Beat-to-beat alternans appeared in RA at BCLs in the range from 650 to 860 ms, as shown in Figure 4L.

Therefore, our simulations show that the KCNH2 T436M, KCNH2 T895M, and KCNE3-V17M mutations reduced the APD and tended to flatten the APDr.

### Effects of the Mutations on Temporal Vulnerability and Stability of Rotors

**FIGURE 5:**
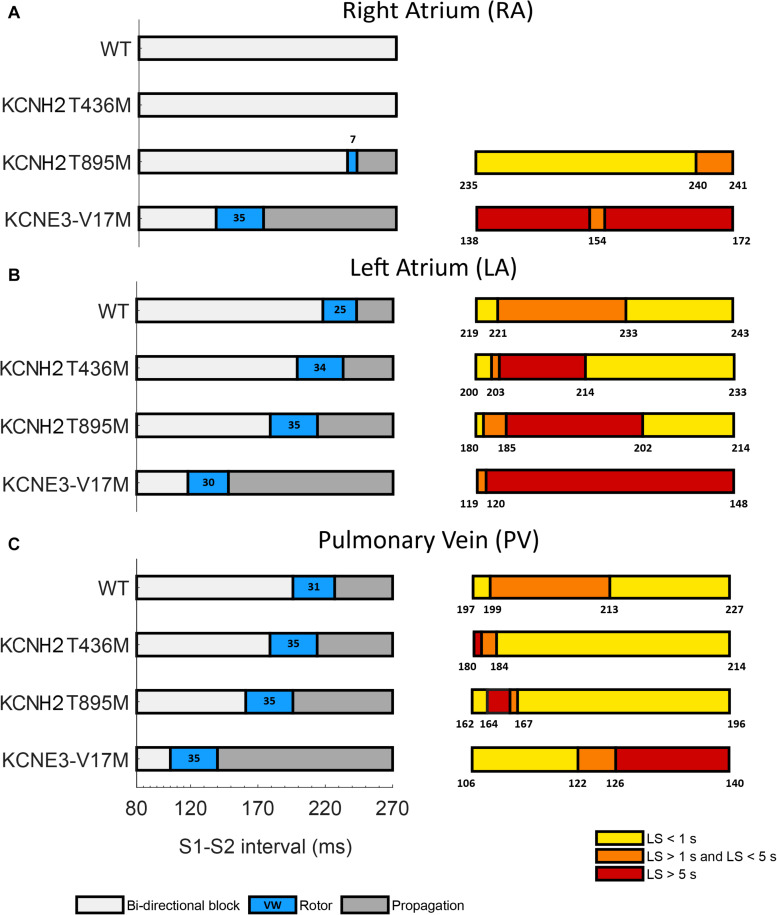
Temporal vulnerability to spiral waves generation of **(A)** right atrium, **(B)** left atrium, and **(C)** pulmonary vein tissues in WT conditions and in the presence of the KCNH2 T436M, KCNH2 T895M, and KCNE3-V17M mutations. Left panels: bi-directional conduction block (white), rotor generation (blue), and propagation (gray). Numbers inside the blue regions (rotor) are the vulnerable window widths in ms. Right panels: classification of the rotors elicited in each VW based on their life-span (LS): smaller than 1 s (yellow), from 1 to 5 s (orange), and longer than 5 s (red). Numbers indicate the boundaries.

Temporal vulnerability to re-entry and phase analysis allowed characterization of the susceptibility to arrhythmia of the three tissue models affected by the three genetic mutations under study. The cross-field protocol was firstly applied to healthy tissue (WT) and then to mutation-affected ones. The left panels of Figure 5 depict S1–S2 intervals leading to bi-directional block (white), the initiation of a rotor (orange) and a simple propagation (gray). The right panels represent a zoom of each VW and provide a color-coded life span of the rotors: dark orange represents the sustained VW, yellow corresponds to rotor life spans within 1 and 5 s and light yellow represents rotors lasting less than 1 s.

In the RA region (Figure 5A), the WT and KCNH2 T436M failed to initiate a re-entry, whilst the mutation KCNH2 T895M was able to induce a rotor in a narrow window of time (width: 7 ms). Almost 71% of rotors in this interval faded in the edge of the tissue before reaching 1 s of life and the remaining 29% lived for a maximum of 1.2 s. KCNE3-V17M instead showed a 35-ms wide VW and 99% of induced rotors sustained during the whole simulation (sustained VW).

LA was the region with the highest sustained VW width (left panel of Figure 5B in red). The computed VW in control was 25 ms wide and 52% of induced rotors had a maximum life span comprised of between 1 and 2.3 s. The tissue with the mutation KCNH2 T436M showed a VW width of 34 ms, 35% of which yielded rotors that were sustained until the end of the simulation. The tissue of the KCNH2 T895M mutation also exhibited a wide interval of time (35 ms) in which the tissue was more vulnerable to re-entries; during this time frame, 51% of the rotors were sustained for the entire simulation time. Lastly, the tissue with KCNE-V17M showed a temporal VW of 30 ms, 99% of which were maintained for more than 5 s (sustained VW).

The PV tissue (Figure 5C) has the highest vulnerability to rotor initiation. Temporal vulnerability of the tissues in the presence of the mutations increased just slightly with respect to the WT tissue. In WT rotors were induced if stimulated in a time interval of 31 ms and 48% of them had a lifetime between 1 and 1.6 s. The mutation KCNH2 T436M slightly increased the VW width in 4 ms, but just 7% (the first two instants of time) of elicited re-entries were perpetuated up to the simulation end (sustained VW). 88% of rotors disappeared at the tissue border before 1 s of life. The VW width of the KCNH2 T895M mutation, was also 35 ms, only 9% of which provoked sustained rotors. Similarly, the vulnerable window in the presence of the KNCE3-V17M mutation was 35 ms, but in this case 43% of the width of the VW presented stable rotors, lasting until the end of the simulation.

Overall, the results showed that the presence of the mutations shifted the VW to shorter S1–S2 time intervals (left panels of Figure 5, orange) and increased the width of sustained VW (right panels of Figure 5, red). The VW shift is more evident for the KCNE3-V17M mutation, while it is progressively less pronounced for the KCNH2 T895M and KCNH2 T436M mutations. Likewise, the KCNE3-V17M impacted tissue vulnerability the most, showing the largest sustained VW widths, followed by the KCNH2 T895M mutation and finally by the KCNH2 T436M mutation. Moreover, temporal susceptibility to the rotor’s initiation was higher in PV tissue, closely followed by LA, but rotors were more easily maintained in LA. The effect of the KCNE3-V17M mutation on the width of VW was similar in the three tissues. The presence of any of these mutations induced and sustained re-entries in LA and PV tissues, contrary to WT conditions.

**FIGURE 6:**
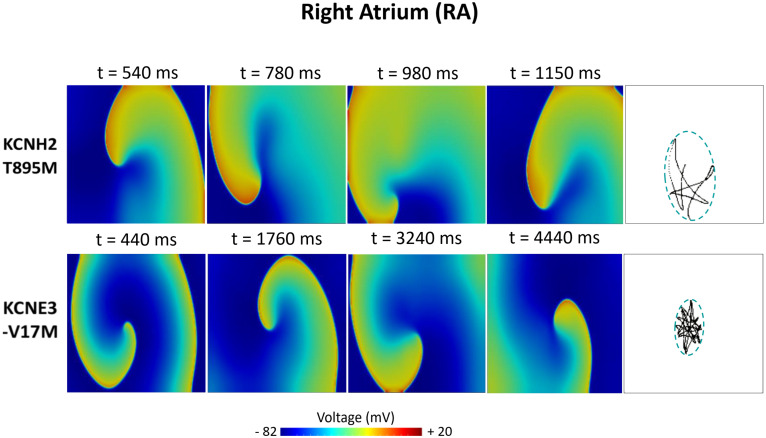
Simulation of spiral waves in 2D right atrium affected by the KCNH2 T895M and KCNE3-V17M mutations. Color-coded voltage snapshots of the rotors and rotors’ tip trajectory enclosed by an ellipse for the quantification of the meandering rotor’s area. S1–S2 interval for KCNH2 T895M model was 241 ms. WT and KCNH2 T436M cases are not shown since no rotors were induced.

**FIGURE 7:**
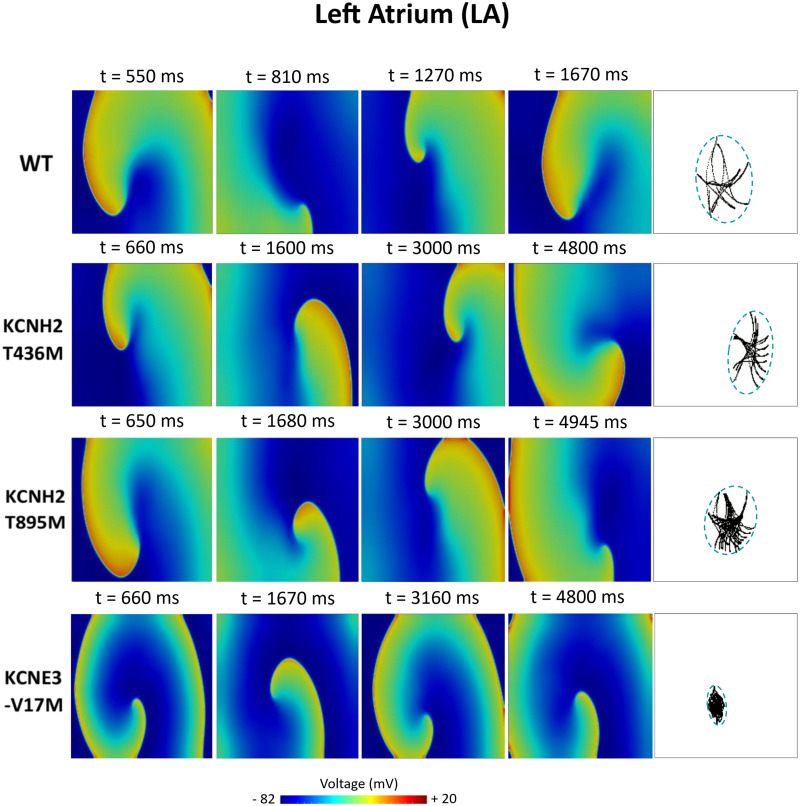
Simulation of spiral waves in 2D left atrium in control (WT) and affected by KCNH2 T436M, KCNH2 T895M, and KCNE3-V17M mutations. Color-coded voltage snapshots of the rotors and rotors’ tip trajectory enclosed by an ellipse for the quantification of the meandering rotor’s area. S1–S2 interval for WT model was 223 ms.

**FIGURE 8:**
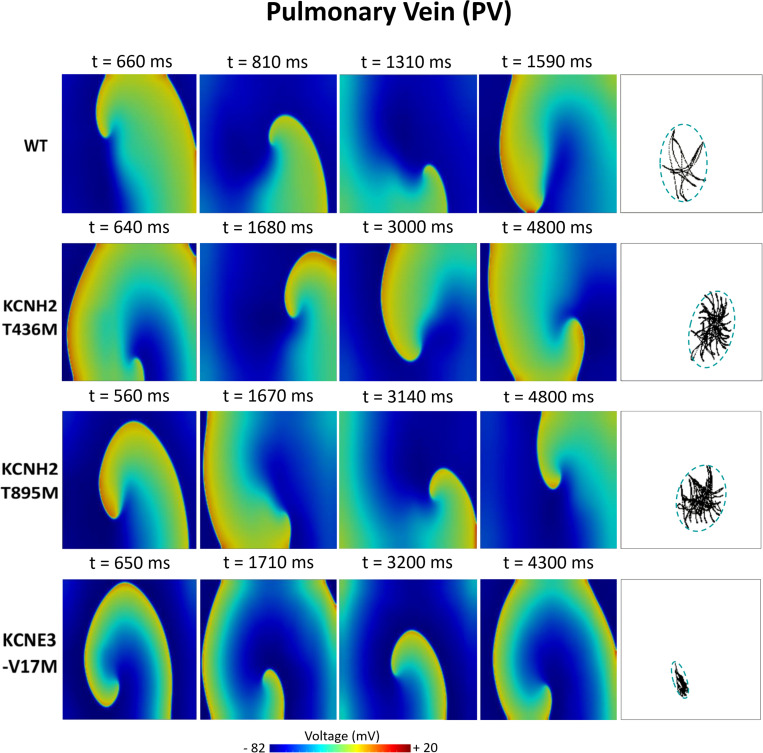
Simulation of spiral waves in 2D pulmonary in control (WT) and affected by KCNH2 T436M, KCNH2 T895M, and KCNE3-V17M mutations. Color-coded voltage snapshots of the rotors and rotors’ tip trajectory enclosed by an ellipse for the quantification of the meandering rotor’s area. S1–S2 interval for WT model was 205 ms.

Figures 6–8 illustrate snapshots of the rotors induced in WT and in the presence of mutations in RA, LA, and PV and the rotor tip’s trajectory, which was enclosed in the ellipse. The rotors shown were elicited with the S2 at the center of the sustained VW, when possible. Otherwise, they were elicited with the S2 yielding the re-entry with the longest possible life span (rotors in LA and PV in WT and in RA in the presence of the KCNH2 T895M). Rotor tip trajectories exhibited a star-shaped path in all cases, except in LA and PV in the presence of the KNCE3-V17M mutation, where the meandering of rotors appeared more circular. Table 3 summarizes the areas of the ellipses (cm^2^) surrounding rotor’s tip trajectories. The smallest areas were registered in PV followed by LA and in the presence of the KCNE3-V17M mutation, followed by the KCNH2 T895M and the KCNH2 T436M mutations. These results highlight the ability of LA and PV tissues to accommodate stable re-entries.

**TABLE 3:** Areas of the ellipses (cm^2^) surrounding rotor’s tip trajectories.

	**RA**	**LA**	**PV**
WT	–	18.95	14.84
KCNH2 T436M	–	14.01	13.99
KCNH2 T895M	18.14	13.26	12.62
KCNE3-V17M	6.69	2.6	1.97

## Discussion

### Main Findings

The simulation study presented in this paper proposes to gain insights into the mechanisms of the AF induced by three gain-of-function mutations—KCNH2 T895M, KCNH2 T436M, and KCNE3-V17M—using 0D and 2D human atrial electrophysiological models. To do so, we first proposed formulations of the potassium currents altered by the mutations (I_*Kr*_ and I_*to*_) which were fitted to experimental data using a genetic algorithm. Then, versions of the Courtemanche et al. (1998) human atrial myocyte model including the acetylcholine current formulation from the Grandi et al. (2011) model were created to simulate the presence of these mutations. In addition, we considered electrical remodeling to account for atrial heterogeneities for RA, LA, and PV. Effects on membrane potential, ionic currents and electrical propagation of the waves in the different atrial tissues in the absence and in the presence of these mutations were analyzed. The main findings are the following: (i) the changes induced by these mutations led to larger I_*Kr*_ and/or I_*to*_; (ii) as a direct consequence, APD_90_ values were abbreviated and APDr curves were flattened; (iii) the three mutations increased tissue vulnerability to re-entry; (iv) the study of the computation of the sustained VW and the areas of the tips allowed the identification of the KNCE3-V17M mutation as the most pro-arrhythmic, followed by the KCNH2 T895M mutation and by the KCNH2 T436M mutation as the least pro-arrhythmic; (v) LA and PV tissues were much more arrhythmogenic than RA, with the LA tissue showing wider sustained VWs and PV tissue revealing the highest temporal susceptibility to the rotor’s initiation.

To the best of our knowledge, this work represents the first computational study of the generation and maintenance mechanisms of AF induced by the three gain-of-function mutations—KCNH2 T895M, KCNH2 T436M, and KCNE3-V17M—on human atrial electrophysiological models at the cellular and tissue levels. Two novel aspects of this work are the study of the effects of the mutation in different atrial tissues and the comprehensive study of the VW, as it was calculated using three criteria based on their life span. They allowed us to observe interesting aspects, such as longer VW to sustained reentry in LA than in PV and that the presence of the KCNE3-V17M mutation led to the greatest prolongation of the VW to sustained reentries in all the atrial tissue types.

Our findings demonstrate pro-fibrillatory effects of these mutations in spiral wave initiation and maintenance, representing a potential risk of developing severe forms of arrhythmia for patients affected by them.

### Pro-arrhythmogenic Effects

APD_90_ reduction is considered a pro-arrhythmogenic index, as it has been associated with the initiation of AF in previous studies (Bosch et al., 1999; Hong et al., 2005a; Loewe et al., 2014). In the present study, the mutations reduced the APD_90_ to different extents and this is linked to higher amplitudes of potassium currents, as explained previously. Similar APD_90_ values were shown in a previous study, where electrical remodeling induced by AF was applied to a version of the Courtemanche model (Colman et al., 2013). The reduction of the APDr maximum slope value was also investigated. In fact, several studies were conducted to assess the causative link between the steepness of the APDr curve and the instability of spiral waves, but the APDr curve slope was not confirmed as the main parameter to determine rotor instability (Cherry and Fenton, 2004; Ten Tusscher and Panfilov, 2006). On the other hand, in our study, a reduction in the maximum slope values of APDr curves was observed in the mutation’s conditions; steep restitution curves were not present, but rotors simulated in 2D tissues exhibited some instabilities, provoking wave breaks. A similar result was reported in a previous study by Kharche et al. (2012), where loss of rate-dependent adaptation was comparable to the ones manifested by patients with chronic AF. In addition, APDr curves in LA and PV in the presence of the studied mutations, and in RA in the presence of the KCNE3-V17M mutation, resemble the trends of those shown by patients suffering from chronic AF in the experimental studies from Franz et al. (1997) and Dobrev and Ravens (2003). Thus, our findings could support the hypothesis that such strong flattening of APDr curve may represent an index of pro-arrhythmogenicity.

Atrial susceptibility was investigated through temporal vulnerability, which indicates the time window when a premature stimulus S2 could trigger the generation of spiral waves. Susceptibility of the atrial tissue was increased by the three mutations under study, as shown by the larger width of VWs, as seen in Figure 5. However, no significant difference was observed in the VW width obtained for re-entries that lasted 2 cycles at least in the LA and PV tissues among mutations, despite their different pro-arrhythmogenicity to sustained re-entries. This result is consistent with a previous study, where a mutation-induced increase of K^+^ current led to slight differences among their VWs (Kharche et al., 2012; Zulfa et al., 2016). In our research, the study of the VW was extended not only on the rotor’s initiation, but also in its ability to be sustained up to the end of the simulation. This fact supports the idea that the VW width for sustained re-entries could provide a better estimation of the impact of the mutations on the susceptibility to arrhythmias. Tissue susceptibility is also linked to spatial vulnerability, which is the critical size of the tissue required to hold a re-entry and make it sustained. This index is related to the wavelength of excitation impulses, which is in turn linked to the duration of the action potential. A decreased APD thus leads to a decreased excitation wavelength, thereby resulting in a reduced dimension of the substrate needed to promote and sustain a re-entry. Indeed, arrhythmias can be more easily maintained since tissues recover faster from refractoriness. Snapshots of the rotors in Figures 6–8 support this explanation. Our observations appear similar to those of a previous work, where the effects of a mutation were studied at different degrees of expression (Kharche et al., 2012). The wide region of tissue occupied by the tip of the rotor in WT conditions and in the presence of the KCNH2 T895M mutation in RA observed in our study resembles the area of the tip of the rotors in WT in their research, where less recovered tissue and a consequent wider meandering area were shown. Similarly, the stronger the effects of the mutations, the smaller the area of rotors’ tip meandering. Moreover, in the present study, the differences in the tip trajectory area observed among the three atrial regions could point to a higher arrhythmogenicity of LA and PV tissues, with respect to RA.

In conclusion, we have shown that abbreviated APD and increased tissue vulnerability to stable re-entries are the effects of the KCNH2 T895M, KCNH2 T436M, and KCNE3-V17M mutations. However, the impact is different: KCNE3-V17M turned out to be the mutation with the highest risk of arrhythmogenicity, and it was even more risky in the LA and RA than in the PV when considering the width of the sustained vulnerable window. Regarding the meandering area of the rotors, the smallest values were registered in the PV region, followed by LA and finally RA. Susceptibility to re-entry in tissues affected by the KCNH2 T895M and KCNH2 T436M mutations was higher—both in terms of sustained vulnerable window width and in terms of the stability of rotors (meandering area of rotors)—in LA than in PV. Moreover, the KCNH2 T895M mutation has a stronger impact than KCNH2 T436M.

### Relevance Respect to Previous Studies

I_*Kr*_ gain-of-function, provoked by genetic mutations affecting the KCNH2 gene, has previously been identified in different cardiac pathologies. The N588K mutation, first reported by Brugada et al. (2004), was found in three unrelated families, with a wide range of phenotypes going from atrial to ventricular fibrillation, short-QT syndrome (Hong et al., 2005a; McPate et al., 2005) and sudden death associated with SQTS (Brugada et al., 2004). Another hERG mutation, L532P, leading to AF was identified by Hassel et al. (2008). Sun et al. (2011) and El Harchi et al. (2012) described a third defect, T618I, also associated with SQTS. Patients affected by these genetic mutations linked to AF presented a shortened action potential and effective refractory period, demonstrating the link between the arrhythmia and the abbreviated APD.

Rare variants affecting the KCND3 gene, coding for Kv4.3, were also associated with lone AF cases with early on-sets. Initially, KCND3 mutations were investigated by Mann et al. (2012) and no effect of cellular electrophysiology was observed. But then, in the first case reported by Olesen et al. (2013), a gain-of-function mutation impaired the transient outward current acting directly on the pore-forming subunit of its channel. The mutation, A545P, produced a shortening in APD and was associated with genesis of AF. Similar results were described in a second mutation in the KCND3 gene (Huang et al., 2017).

The KCNE is a family of voltage-gated potassium channel accessory subunits composed of 5 members, and it modulates specific potassium currents and plays a role in arrhythmias (Crump and Abbott, 2014). Some genetic defects on these accessory subunits have been associated with lone atrial fibrillation, as reported in previous studies for KCNE1 (G25V and G60 mutations) (Olesen et al., 2012), and for KCNE2 (R27C, M23L, and I57T mutations) (Yang et al., 2004; Nielsen et al., 2014). Their results associate the presence of such mutations with increased susceptibility to AF. In addition, variants of the gene KCNE3 were associated with arrhythmogenic outbreak when expressed in the Kv11.1 channel complex (Lundby et al., 2008).

Our simulation study identifies, at different scales, important changes in atrial fibrillation biomarkers and supports the hypothesis that potassium-channel-related mutations could lead to the generation of a substrate more susceptible to arrhythmic events. Many computational studies have reproduced the effects of genetic mutations affecting potassium channels (Kharche et al., 2008, 2012; Imaniastuti et al., 2014; Loewe et al., 2014; Zulfa et al., 2016) and have demonstrated that abbreviated APD and wavelength, as well as increased tissue vulnerability (similarly to our findings), are proarrhythmic factors. Loewe et al. (2014), in particular, demonstrated by simulating the effects of mutations on KCNH2 gene that APD and effective refractory period shortening combined to a more linear repolarization phase (triangular AP) were alone sufficient to induce a substrate for AF. The effects of these mutations was further investigated by extending that work to 3D anatomy with AF electrical remodeling, where the arrhythmogenic potential of the KCNH2 L532P and N588K mutations was confirmed (Heikhmakhtiar et al., 2020).

Together with previous computational studies (Moreno et al., 2013; Cano et al., 2020), our investigation could represent a step forward in the development of personalized anti-arrhythmic drugs based on genotype characteristics. Furthermore, these patient-specific models can provide useful insights not only in improving diagnosis and in guiding therapeutical plans, but also in prevention and management of AF by early identification of patients at risk of developing AF later in life.

## Limitations

The mutations’ effects were investigated using the Courtemanche et al. (1998) cardiac model, which is widely used in atrial simulations and provides a valid and accurate tool with reduced complexity. Although we would expect to obtain similar effects on the main AF biomarkers studied in this work, the assessment of the mutant effects with other mathematical models would indeed improve the evaluation of the mutations’ arrhythmogenic potential on currents such as sodium and calcium, which are better characterized by other frameworks.

The patients affected by the genetic defects analyzed in this study carried the heterozygous form of the mutations. However, experimental data were provided only in homozygous conditions. Thus, our simulations reproduce a condition less likely to happen. Some theoretical studies simulate the heterozygous conditions by exposing only half of the channels to the mutation’s effects. However, in many cases, heterozygous expression of mutations do not really differ this much from the homozygous ones, as demonstrated by the dominant effect of Kv4.3/KCNE3-V17M in Lundby et al. (2008). Therefore, we chose to investigate the cases with highest risk to understand the severe forms of the AF episodes probands are subjected to.

The present study merely characterized the pro-arrhythmic effects of the genetic mutations on atrial electrophysiology. The electrical and structural changes induced by AF are also likely to contribute in the onset and perpetuation of the arrhythmia (Dössel et al., 2012), thus the study of their effects on atrial currents and structures together with the presence of the mutations would add further knowledge to the long-term effects of such pathologies.

Gender-related changes in phenotype in the presence of the same mutations should be explored since the same mutation can lead to different symptoms. The KCNE3-V17M mutation in this work was also carried in fact by the proband’s sister. However, the symptoms manifested were different as she never suffered any AF. Possible explanations from the author of the original study are that this difference may result from environmental factors or is simply a reflection of a low penetrance (Lundby et al., 2008). On the contrary, the KCNH2 T436M mutation was carried also by the proband’s sister, affected with AF since a young age (Hayashi et al., 2015). The study and characterization of the electrophysiological experiments on female individuals could be useful to have a more complete understanding of the underlying properties of such mutations.

The computational study hereby presented was performed at a single-cell and two-dimensional tissue scale. A further extension of our investigation to three-dimensional realistic atrial geometries would allow completing and validating our results at the whole-heart level as well.

Nevertheless, we do not expect these limitations to alter the main conclusions of this work.

## Data Availability Statement

The original contributions presented in the study are included in the article/Supplementary Material, further inquiries can be directed to the corresponding author/s.

## Author Contributions

RB, LR, LM-M, and JS conceived, designed the study, and analyzed the results. FF and EC provided the original optimization algorithm. RB adapted the previous code to the study, implemented new features, performed the optimization procedures and the simulations, and wrote the first draft of the manuscript. All authors revised and approved the manuscript.

## Conflict of Interest

The authors declare that the research was conducted in the absence of any commercial or financial relationships that could be construed as a potential conflict of interest.

## Supplementary Material

Supplementary Figure 1 Sensitivity analysis for KCNH2 T436M and KCNH2 T895M I_*Kr*_ current formulation.

Supplementary Figure 2 Sensitivity analysis for KCNE3-V17M mutated Kv11.1 channel complex.

Supplementary Figure 3 Sensitivity analysis for KCNE3-V17M mutated Kv4.3 channel complex.
