# Supplementary material for: Arrhythmogenic Effects of Genetic Mutations Affecting Potassium Channels in Human Atrial Fibrillation: A Simulation Study
Source: Front Physiol. 2021 May 31;12:681943. doi: 10.3389/fphys.2021.681943 (PMC8201780; doi:10.3389/fphys.2021.681943)
Supplement: Supplementary Figure 1 — Sensitivity analysis for KCNH2 T436M and KCNH2 T895M IKr current formulation. [file Data_Sheet_1.docx]

**SUPPLEMENTARY MATERIAL**


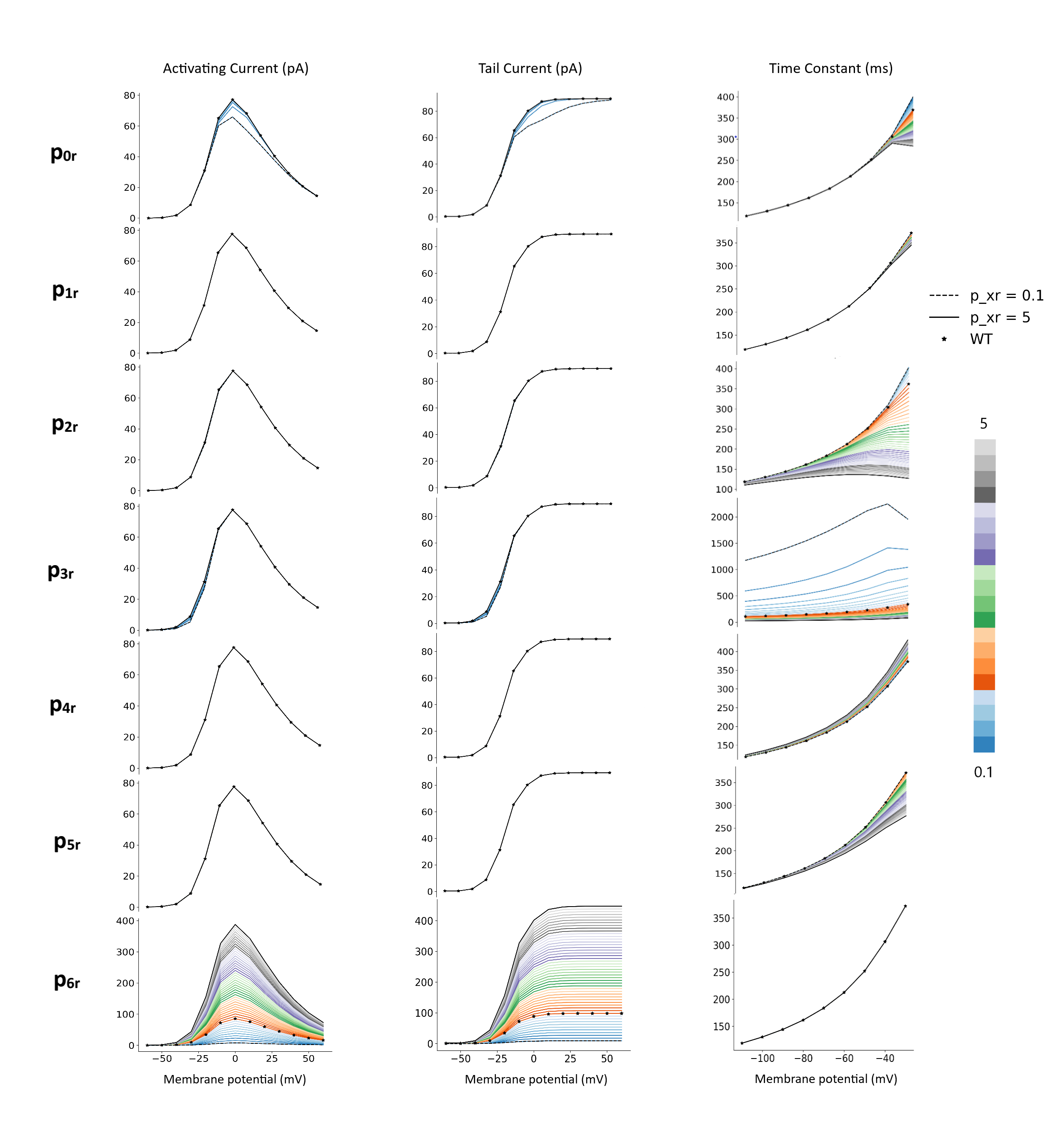


Figure 1S. Sensitivity analysis for KCNH2 T436M and KCNH2 T895M I_Kr_ current formulation. Changes in activation current, tail current and deactivation time constant caused by variations on parameters’ values, in the range from 0.1 to 5, colour-coded as shown by the bar. Parameters p_0r_ to p_6r_ are defined in Equations 1-3.


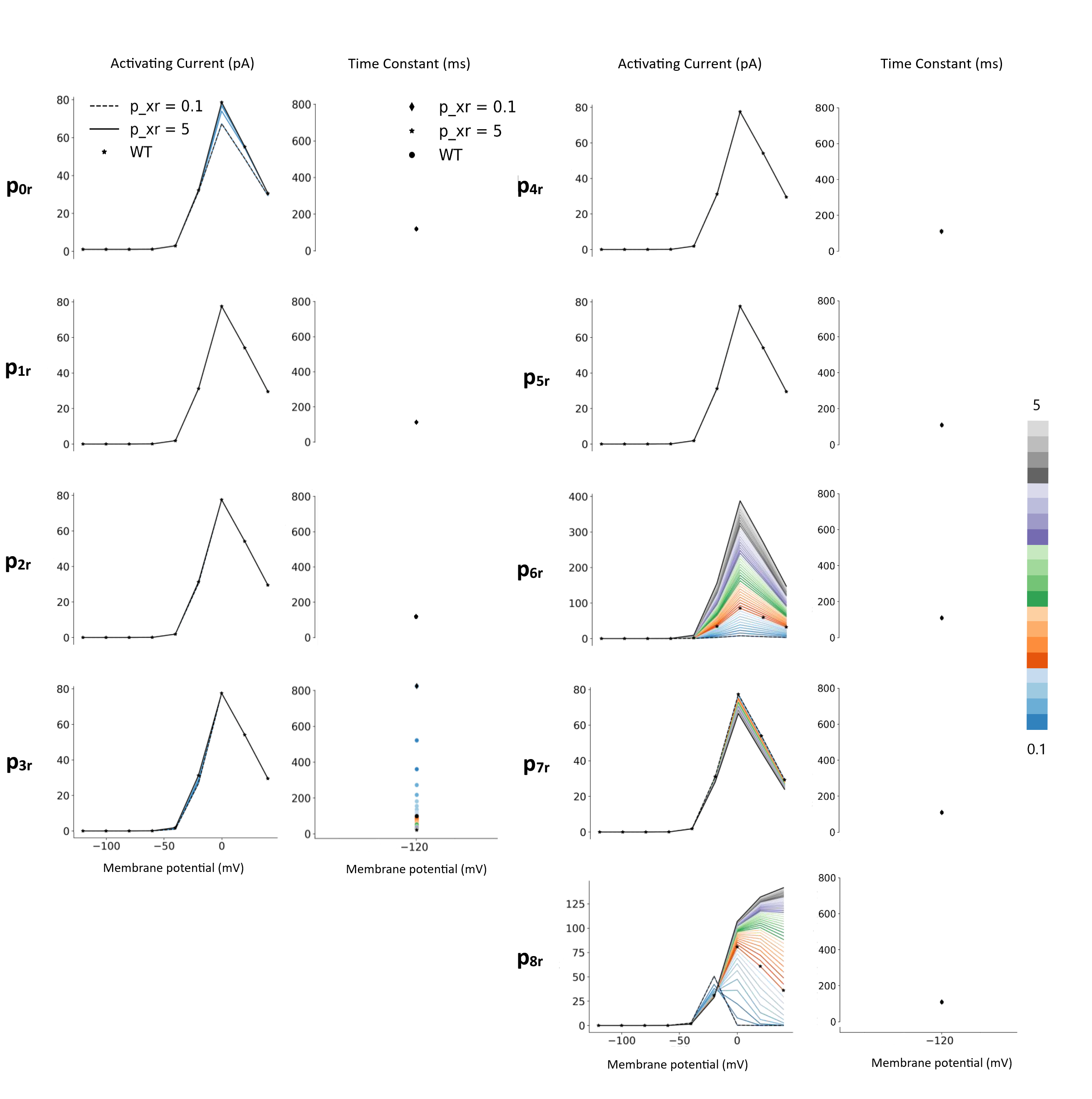


Figure 2S. Sensitivity analysis for KCNE3-V17M mutated Kv11.1 channel complex. Changes on I-V relationship (left) and deactivation time constant at -120 mV (right) caused by variations on parameters’ values, in the range from 0.1 to 5, colour-coded as shown by the bar. Parameters p_0r_ to p_8r_ are defined in Equations 1-3.


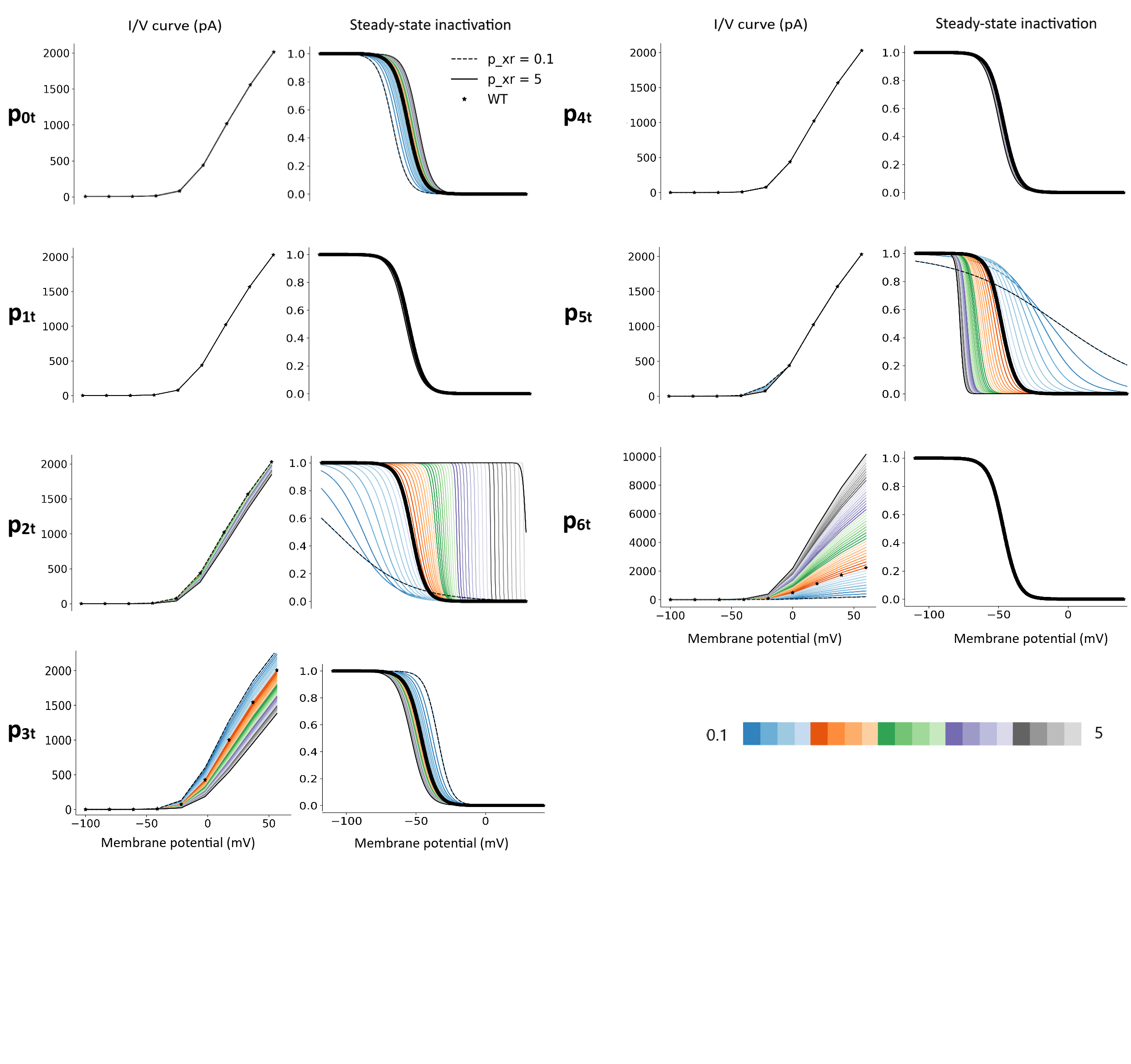


Figure 3S. Sensitivity analysis for KCNE3-V17M mutated Kv4.3 channel complex. Changes on I-V relationship and steady-state inactivation caused by variations on parameters’ values, in the range from 0.1 to 5, colour-coded as shown by the bar. Parameters p_0t_ to p_8t_ are defined in Equations 4-6.
